# Photodynamic activation of a KRAS RNA G-quadruplex–targeted photosensitizer induces ferroptosis in cisplatin-resistant non–small cell lung cancer

**DOI:** 10.1016/j.jbc.2026.111181

**Published:** 2026-01-20

**Authors:** Xiao-Dong Wang, Jia-Hong Lin, Ming-Hao Hu

**Affiliations:** Nation-Regional Engineering Lab for Synthetic Biology of Medicine, International Cancer Center, School of Pharmacy, Shenzhen University Medical School, Shenzhen, China

**Keywords:** NSCLC, KRAS, RNA G-quadruplex, multifunctional, ferroptosis

## Abstract

KRAS overactivation plays a crucial role in the development of non–small cell lung cancer (NSCLC). Although two KRAS inhibitors have been approved for NSCLC treatment, their efficacy is limited to the KRAS G12 C mutant along with the occurrence of drug resistance. Previously, we discovered a small molecule (**MBD**) targeting RNA G-quadruplex (RG4) in the 5′-UTR of KRAS mRNA. However, **MBD** exhibited some side effects, owing to the presence of KRAS RG4 in normal cells. Hence, there is a need for innovative strategies to mitigate the side effects associated with KRAS RG4-targeted ligands. In this study, we paid attention to photodynamic therapy (PDT), and thus a new photosensitizer termed **MC1** was discovered. **MC1** displayed considerable binding capacity and selectivity to KRAS RG4, compared to other RNAs. Photosensitivity of **MC1***in vitro* was illustrated by KRAS RG4 breakage, GSH consumption, and NADH oxidation, leading to the occurrence of ferroptosis in cisplatin-resistant NSCLC cells. Antitumor efficacy of **MC1** was verified in A549/DDP-bearing nude mice. Moreover, **MC1** demonstrated superior performance than **MBD** in several aspects, including G4 stabilizing ability *in vitro*, KRAS inhibitory efficacy at the cellular level, and drug safety at the animal level. Besides, the potential of **MC1** as a fluorescent probe for KRAS RG4 was characterized. To sum up, our study provides guidance for the development of KRAS RG4-targeted photodynamic therapy strategies for the treatment of NSCLC.

Non–small cell lung cancer (NSCLC) accounts for 85% of the lung cancer with high morbidity and mortality. Although therapeutic strategies for tumor develop rapidly in the recent years, only platinum-based chemotherapy and immunotherapy are in clinical application for the patients of advanced-stage tumor (1, 2). Furthermore, drug resistance emerges and becomes a huge challenge for the treatment of NSCLC (3). RAS proteins, including KRAS, HRAS, and NRAS, are implicated in a variety of physiological responses, and cancer cells with RAS mutations usually display more aggressive phenotypes (4). KRAS amplification is often present in solid tumors, accounting for 78% of all RAS mutations in NSCLC (5). KRAS mutant subtypes are primarily classified as G12D, G12V, G12C, G12R, G12A, and G13D mutations, along with KRAS wildtype amplification (6). Despite the identification of KRAS as a common driver of oncogenesis, it has long been considered as undruggable due to the absence of suitable binding sites within its mutant isoforms, until FDA approves two KRAS G12C inhibitors (sotorasib and adagrasib) for advanced solid tumors in 2021 and 2022 (7). MRTX1133, the first noncovalent and selective inhibitor for KRAS G12D, demonstrates anticancer properties and enters clinical trials in 2024. Pan/multi-RAS/KRAS inhibitors are also proved to prevent the activation of a broad range of KRAS mutants in preclinical phase (8, 9). Even though advancements are exciting for KRAS-targeted therapies, there are still challenges to upgrade clinical response rate and avoid drug resistance. Moving forward, it is crucial to investigate small molecule inhibitors, especially blocking KRAS pathways *via* novel targets and mechanisms.

In these years, RNA G-quadruplexes (RG4s), located in 5′-untranslated region (5′-UTR) of mRNA, attract increasing attention in anticancer strategies, since RG4s are commonly with high stability due to their G-tetrads by Hoogsteen base paring, which can be interacted with protein factors or nucleic acids (10, 11). Several types of oncogenic RG4s are proved to be essential in preventing the translation of mRNA, like BCL-2, NRAS, and KRAS (12, 13). Human KRAS transcript contains a 5′-UTR sequence of 192 nucleotides with a GC content of 77%, and three nonoverlapping G4 motifs can be formed, given that a G4 motif is composed by two G-tetrads and loop length up to 12 nt. Small molecule ligands with the ability to target KRAS RG4 are proved to efficiently repress KRAS protein levels (14). Novel coumarin-quinolinium derivatives are designed and identified as pan-KRAS translation inhibitors by targeting KRAS RG4, exhibiting antiproliferative activity against cancer cells through MAPK and PI3K-AKT pathways (15). In addition, a tribenzophenazine analog (**MBD**) with the same target is reported by our group, to be an effective therapeutic agent for KRAS-driven NSCLC with chemotherapy resistance (16). However, during our study, we observed that **MBD** also triggered considerable cytotoxicity on normal cells, ultimately resulting in weight loss at a higher dosage, which may be due to the existence of KRAS RG4 in normal cells. Therefore, novel strategies should be implemented to attenuate the side effects of KRAS RG4-targeted ligands.

Photodynamic therapy (PDT) emerges as a clinically useful means to treat cancers, owing to its notable advantages, such as high selectivity, excellent tolerability, and minimal invasiveness (17). Upon irradiation, photosensitizers are excited from ground state (S_0_) to singlet state (S_1_) and then to triplet state (T_1_). The energy release from T_1_ rapidly back to S_0_ transfers O_2_ into reactive oxygen species (ROS), and the local ROS in the focused area is capable of triggering tumor cell death, sealing blood vessels, and so on (18, 19). Until now, photosensitizers in clinical use are predominantly porphyrin-based reagents, for example the first commercialized one, porfimer sodium, employed in the treatment of solid tumors. Nonporphyrin structures need to be discussed, due to some shortcomings of porphyrins, like weak photostability and long-lasting skin phototoxicity (20). Besides, the efficacy of PDT may become compromised as tumor cells upregulate antioxidant proteins to reduce intracellular ROS levels. Given that G4s are susceptible to be oxidized, a few photosensitizers targeting G4s have been reported. A derivative of a typical G4 ligand (TMPyP4) with the structure of porphyrin binds to KRAS RG4 and represses KRAS translation upon photoactivation (21). Other types of small molecules targeting RG4s are designed with PDT potential, eliciting augmented immunity for cancer elimination (22, 23). Nevertheless, the number of G4-targeted photosensitizers is rather limited, and their anticancer mechanisms are superficially evaluated, leaving an urgent need to discover novel scaffolds and mechanisms.

We then turned our attention to ferroptosis, a form of regulated cell death, which can be triggered by the increase of intracellular oxidative status. In addition, Fenton reaction during ferroptosis between H_2_O_2_ and ferric ion produces more O_2_. Thus, ferroptosis and PDT can foster mutual enhancement to achieve antitumor potential (24, 25). Our previous study proved the dibenzophenazine core of **MBD** as an excellent scaffold for photosensitizers. Nevertheless, **MBD** only displayed modest photo-induced cytotoxicity, without the potential as a turn-on fluorescent probe for labeling G4s. Informed by our expertise in boosting both ROS generation and G4-responsive fluorescence in compounds, we substituted the benzene ring in **MBD** with a piperazine ring, resulting in the new ligand **MC1** in this study. Binding property and selectivity of **MC1** to KRAS RG4 were first determined by absorption and fluorescence spectroscopy *in vitro*, and the potential of **MC1** as a fluorescent probe was further confirmed in A549/DDP cells, a human NSCLC cell line resistant to cisplatin (DDP). Photosensitivity of **MC1** was displayed both *in vitro* and in tumor cells, including ROS production, KRAS RG4 breakage, GSH consumption, and NADH oxidation. Phototoxicity of **MC1** against A549/DDP cells was then investigated, especially with regard to ferroptosis-related pathways. Finally, **MC1** was proved to be effective for fluorescent imaging and PDT in A549/DDP-bearing nude mice. Our study emphasizes the promise of developing KRAS RG4-targeted PDT strategies for precision cancer therapy.

## Results and discussion

### Design and synthesis of MC1

**Figure 1:**
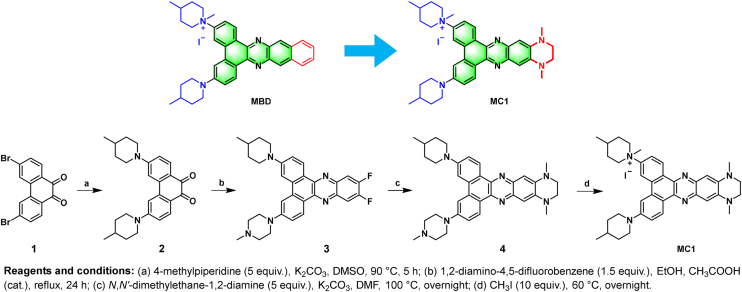
**Design and synthesis of MC1**.

The dibenzophenazine core of **MBD** had been demonstrated to be an excellent scaffold for the construction of photosensitizes by our group (26). Herein, we showed that the IC_50_ value (photocytotoxicity determined by CCK8 assays) of **MBD** upon irradiation at 495 nm (corresponding to its major absorption band, 12.5 mW/cm^2^ for 15 min) was 0.52 ± 0.02 μM, which was 10% of its dark-cytotoxicity, demonstrating modest photo-induced cytotoxicity as well as a low phototoxic index. Besides, **MBD** displayed no enhanced fluorescence upon binding to KRAS RG4 (Fig. S1), rendering it unable to be a turn-on fluorescent probe for labeling G4s. Therefore, we focused on the structural modification of **MBD**. Based on our experience in enhancing ligand's ROS generation ability as well as G4-triggering fluorescence (27, 28), we replaced the benzene ring with a piperazine ring in the structure of **MBD**, ultimately yielding **MC1**. The synthesis of **MC1** was described in Figure 1 (the details were provided in the experimental section), where the synthesis of intermediates **2** and **3** was according to our previous study (16). The structure and purity of **MC1** were confirmed by ^1^H NMR, ^13^C NMR, HRMS, and HPLC spectroscopy.

## Binding property of MC1 to KRAS RG4

**Figure 2:**
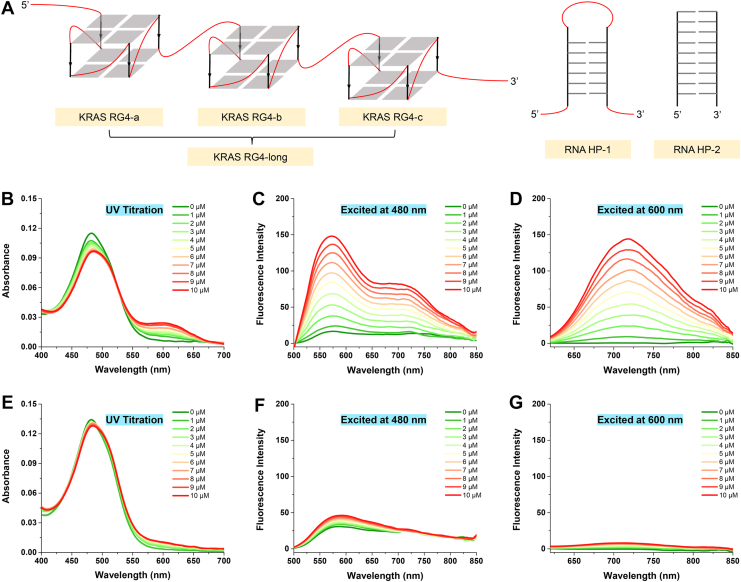
**Binding property of MC1 to KRAS RG4.***A,* structure diagram of KRAS RG4s and RNA HPs used in this study. *B,* UV-vis absorption spectra of 5 μM **MC1** titrated with KRAS RG4-a from 0 to 10 μM. *C* and *D,* fluorescence spectra of 5 μM **MC1** titrated with KRAS RG4-a from 0 to 10 μM, excited at 480 nm or 600 nm. *E,* UV-vis absorption spectra of 5 μM **MC1** titrated with RNA HP-1 from 0 to 10 μM. *F* and *G,* fluorescence spectra of 5 μM **MC1** titrated with RNA HP-1 from 0 to 10 μM, excited at 480 nm or 600 nm. RG4, RNA G-quadruplex.

Structures of KRAS RG4s and RNA HPs used in this study were demonstrated in Figure 2*A*. KRAS RG4-a, KRAS RG4-b, and KRAS RG4-c were three nonoverlapping G4 motifs located within the first 80 nt of KRAS 5′-UTR, and KRAS RG4-long was a full-length RG4 including those three G4 motifs. RNA HP-1 and RNA HP-2 were two hairpin structures with different sequences. Absorption titration assay was first performed to explore the binding property of **MC1** to KRAS RG4. As shown in Figure 2*B*, **MC1** displayed a major band centered at 480 nm, while the gradual addition of KRAS RG4-a decreased the absorption band at 480 nm with a small redshift and increased the absorption band at 600 nm. The appearance of isochromatic point also inferred the strong interaction between **MC1** and KRAS RG4. Meanwhile, the absorption spectra of **MC1** slightly changed with the presence of RNA HP-1 (Fig. 2*E*). As for the fluorescent response toward KRAS RG4, when excited at 480 nm, **MC1** exhibited intensified emission bands centered at 575 nm and 725 nm upon binding to KRAS RG4 (Fig. 2*C*). Similarly, when excited at 600 nm, **MC1** displayed an emission band centered at 725 nm triggered by KRAS RG4 (Fig. 2*D*). With the addition of RNA HP-1, there was little change in the fluorescence spectra of **MC1**, excited at either 480 nm or 600 nm (Fig. 2, *F* and G). CD melting assay was additionally performed, with the results displayed in Figure S2. **MBD** and **MC1** elevated Δ*T*_m_ values of KRAS RG4 for 12 °C and 21 °C, respectively, suggesting a stronger stabilizing ability of **MC1** than **MBD** on KRAS RG4. Therefore, the high, dual-channel fluorescence enhancement of **MC1** made it possible to be a fluorescent ligand for KRAS RG4, which was distinguished from **MBD**.

### Binding selectivity of MC1 to KRAS RG4

**Figure 3:**
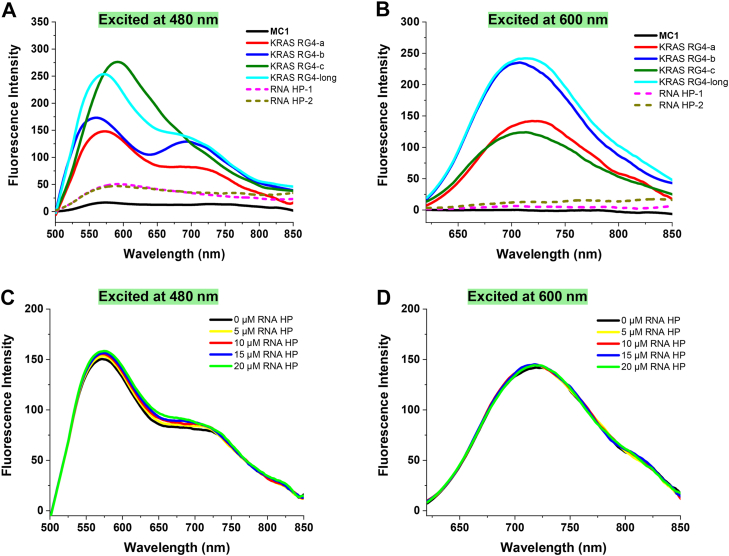
**Binding selectivity of MC1 to KRAS RG4.***A, B,* fluorescence spectra of **MC1** (5 μM) and different types of RNA samples (10 μM), excited at 480 nm or 600 nm. *C* and *D,* fluorescence spectra of 5 μM **MC1** and 10 μM KRAS RG4-a, added with RNA HP-1 from 0 to 20 μM, excited at 480 nm or 600 nm. RG4, RNA G-quadruplex.

Binding selectivity of **MC1** between KRAS RG4s and RNA hairpins (structure diagram displayed in Fig. 2*A*) was further determined by fluorescence spectroscopy. As shown in Figure 3*A*, two intensified emission bands centered around 575 nm and 725 nm emerged, when **MC1** was excited at 480 nm and bound to KRAS RG4-long, KRAS RG4-a, or KRAS RG4-b. **MC1** displayed a major emission band centered at 600 nm upon binding to KRAS RG4-c. On the other hand, fluorescence intensity was quite low for the addition of RNA hairpins, either RNA HP-1 or RNA HP-2. When excited at 600 nm, **MC1** demonstrated significant emission bands centered around 725 nm upon binding to all types of KRAS RG4s, rather than RNA hairpins (Fig. 3*B*). Fluorescence competitive assay was further performed to prove the binding selectivity, where **MC1** and KRAS RG4-a were incubated at 1:2 ratio, with the addition of RNA HP-1 from 1 to 4 M equivalents. As shown in Figure 3, *C* and D, RNA HP-1 exerted little impact on the fluorescence spectra of **MC1** and KRAS RG4-a, indicating the selectivity of **MC1** to KRAS RG4s than RNA hairpins. Selectivity of **MC1** to KRAS RG4 than other RG4s, especially NRAS RG4, was discussed by fluorescence spectroscopy. Although the signal of **MC1** binding to KRAS RG4-a was similar with NRAS RG4, the highest fluorescence intensity was recorded of **MC1** binding to KRAS RG4-long (Fig. S3). We supposed that the selectivity of **MC1** to KRAS RG4 could be due to specific G4 structures of KRAS mRNA. Most of the other RG4s only has one G4 motif, while KRAS RG4 has three nonoverlapping G4 motifs, which could be more efficient and effective for the compound to target.

### Binding of MC1 to KRAS RG4 in tumor cells

**Figure 4:**
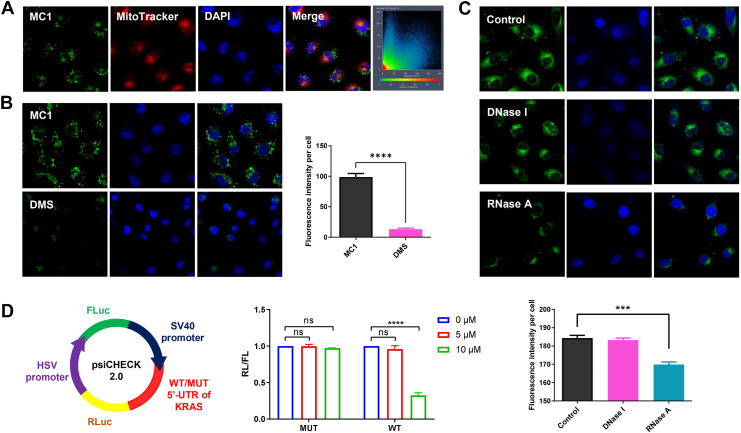
**Binding of MC1 to KRAS RG4 in tumor cells.***A,* Live A549/DDP cells stained with **MC1** (2 μM), MitoTracker Red and DAPI. *B,* Live A549/DDP cells stained with **MC1** (2 μM), with or without the pretreatment of DMS. *C*, Fixed A549/DDP cells stained **MC1** (2 μM), with or without the pretreatment of DNase I or RNase A. *D,* Relative ratio of Renilla luciferase activity to Firefly luciferase activity in psiCHECK-2 vector containing mutant or wild-type 5′-UTR of KRAS, without or with the presence of **MC1**. The data represent the mean ± SD (n = 5), ∗∗∗ for *p* < 0.001, ∗∗∗∗ for *p* < 0.0001 compared to the control group. RG4, RNA G-quadruplex; FL, Firefly luciferase; RL, Renilla luciferase.

To determine the imaging behavior and localization of **MC1** in the cells, live A549/DDP cells were stained with **MC1**, MitoTracker Red, and DAPI and observed by confocal microscopy, with the results displayed in Figure 4*A*. **MC1** emitted bright green fluorescence around the cell nuclei, which did not overlap with the mitochondria, indicating that **MC1** could easily penetrate the cell membrane and accumulate in the cytoplasm. However, the green fluorescence of **MC1** dramatically decreased, when live A549/DDP cells were preincubated with dimethyl sulfate (DMS) (Fig. 4*B*). Since DMS is able to methylate the N7 atoms of the guanine residues to prevent of the formation of G4s (29), the binding target of **MC1** could be G4s in the cytoplasm. Nuclease digestion assay was then carried out to determine whether the target G4 structures were mitochondrial DG4s or cytoplasmic RG4s. As shown in Figure 4*C*, while DNase I exerted no effect, the green fluorescence of **MC1** almost completely quenched after RNase A digestion. Thus, **MC1** may selectively bind to KRAS RG4 in the cytoplasm and illuminate the tumor cells.

To further clarify whether **MC1** can bind to KRAS RG4 in cell models, dual-luciferase reporter assay was performed using PsiCHECK-2 vector, in which the experimental reporter Renilla luciferase (RL) and the control reporter Firefly luciferase (FL) are driven by SV40 promoter and HSV promoter, respectively (14). A wildtype KRAS 5′-UTR sequence (WT) was inserted after the SV40 promoter, as well as a mutant one with no potential to form G4s (MUT), with sequences shown in Table S2. 10 μM **MC1** distinctly declined RL/FL activity in the WT group, while RL/FL activity in the MUT group was not influenced by **MC1** (Fig. 4*D*). Therefore, binding of **MC1** to KRAS RG4 could suppress the translation of KRAS with specificity in the cells.

### Photosensitivity of MC1 *in vitro*

**Figure 5:**
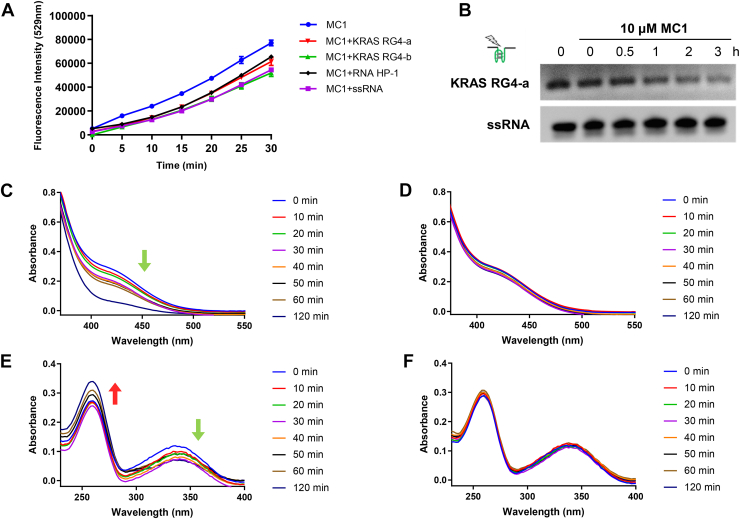
**Photosensitivity of MC1 *in vitro*.***A,* ROS production of 10 μM **MC1** irradiated with 495 nm, 12.5 mW/cm^2^ for different time points, with or without the presence of 10 μM RNA samples, examined by DCFH. *B,* breakage of KRAS RG4 or ssRNA (10 μM) induced by **MC1** (10 μM) as well as irradiation (495 nm, 12.5 mW/cm^2^) for different time points, examined by EMSA. *C,* GSH consumption of 20 μM **MC1** irradiated with 495 nm, 12.5 mW/cm^2^ for different time points. *D,* GSH consumption of 20 μM **MC1** without irradiation. *E,* NADH oxidation of 20 μM **MC1** irradiated with 495 nm, 12.5 mW/cm^2^ for different time points. *F,* NADH oxidation of 20 μM **MC1** without irradiation. RG4, RNA G-quadruplex; ROS, reactive oxygen species.

ROS production was investigated to uncover the possibility of **MC1** as a photosensitizer using 2′,7′-dichlorodihydrofluorescein (DCFH) probe, which could be oxidized by ROS to 2′,7′-dichlorofluorescein (DCF) with green fluorescence. As shown in Figure 5*A*, upon irradiation, fluorescence intensity of DCF triggered by **MC1** increased in a time-dependent manner. Although the addition of different types of RNA samples (KRAS RG4-a, KRAS RG4-b, RNA HP-1, or ssRNA) exerted some negative impacts, ROS levels were still quite high to be effective. Whether ROS could break KRAS RG4 was then determined, since **MC1** with potent photosensitivity could selectively targeted KRAS RG4. CD spectroscopy was applied to examine the effects of G oxidation on KRAS RG4, with the results shown in Figure S4. The positive peak of **MC1** and KRAS RG4-a around 265 nm declined to some extent under the irradiation. That is to say, irradiation led to the possibility of G oxidation, partially influencing the formation of G4 structures. From the results of electrophoretic mobility shift assay, the intensity of KRAS RG4-a treated with **MC1** remarkably decreased, as the irradiation time increased. Meanwhile, ssRNA level remained stable under the same conditions, suggesting that **MC1**/irradiation selectively resulted in the photocleavage of KRAS RG4 (Fig. 5*B*).

GSH is an endogenous bioreductive molecule, with the ability to scavenge ROS and hinder the therapeutic effects (30). GSH levels were determined *in vitro* using 5,5′-dithiobis-(2-nitrobenzoic acid), with the reaction product displayed the absorption peak at 412 nm. As shown in Figure 5*C*, absorbance declined with an increase of irradiation time, especially at 412 nm, suggesting that **MC1**/irradiation could consume GSH. In contrast, GSH consumption could not be detected of **MC1** without irradiation (Fig. 5*D*). NADH as a cofactor plays important roles in regulating redox balance and energy production. Photocatalytic efficiency of **MC1** was decided by absorption spectroscopy, with the results displayed in Figure 5, *E* and F. Absorbance at 340 nm decreased with an increase of irradiation time, while absorbance at 260 nm increased. Absorption of nonilluminated **MC1** exhibited no obvious changes, inferring that **MC1** could oxidize NADH into NAD^+^ upon irradiation. These findings suggested that **MC1** was an excellent photosensitizer, which may be utilize to photo-oxidize biomolecules.

### Antiproliferation effects of MC1 in tumor cells

**Figure 6:**
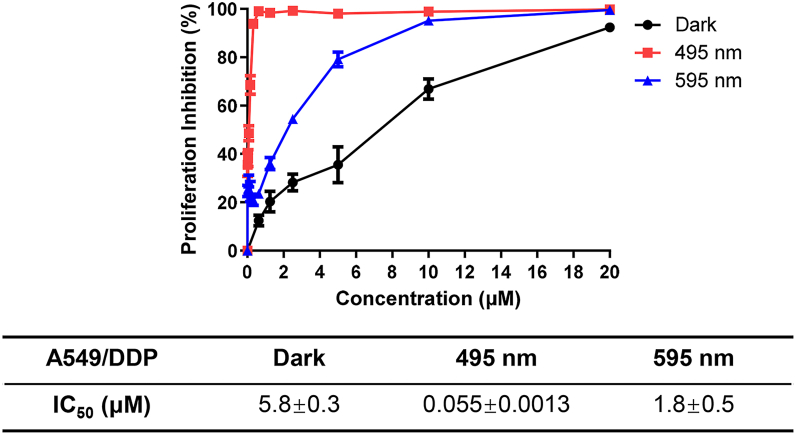
**Cytotoxicity of MC1 against A459/DDP cells, without or with irradiation (495 nm or 595 nm), examined by CCK-8**.

Phototoxicity of **MC1** was determined in A549/DDP cells with the 495 nm and 595 nm LED lights (12.5 mW/cm^2^ for 15 min) corresponding to the two absorption bands of **MC1** with KRAS RG4. As shown in Figure 6, dark-cytotoxicity of **MC1** was relatively lower with the IC_50_ value of 5.8 ± 0.3 μM, which was similar to that of **MBD** in the dark (4.8 ± 0.4 μM), showing this modification may not weaken the cytotoxicity. Notably, the IC_50_ value of **MC1** upon irradiation at 495 nm was below 1% of dark-cytotoxicity (0.055 ± 0.0013 μM), demonstrating an excellent phototoxicity as well as a promising phototoxic index of **MC1**. However, the IC_50_ value of **MC1**, when irradiated at 595 nm, was determined to be 1.8 ± 0.5 μM, which was comparable in magnitude to its dark-cytotoxicity. Thus, irradiation at 495 nm, 12.5 mW/cm^2^ for 15 min was used for the following experiments.

**Figure 7:**
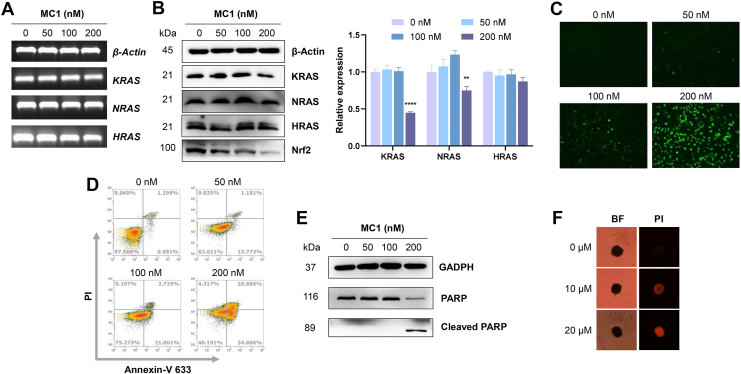
**Antiproliferation effects of MC1 in tumor cells.***A* and *B,* transcription and expression of RAS-related genes in A549/DDP cells after 24-h treatment (**MC1** for 1 h, irradiation with 495 nm, 12.5 mW/cm^2^ for 15 min, and culture for another 24 h), examined by RT-PCR and Western blot. *C*, ROS levels of A549/DDP cells after 24-h treatment, examined by DCFH-DA. *D* and *E,* apoptosis of A549/DDP cells after 24-h treatment, examined by Annexin V-PI staining and Western blot. *F,* growth inhibition of A549/DDP cells after 7-days treatment, examined by MCTS and PI staining. The data represent the mean ± SD (n = 3), ∗∗ for *p* < 0.01, ∗∗∗∗ for *p* < 0.0001 compared to the control group.

Since **MC1** could selectively target KRAS RG4, the transcription and translation of RAS-related genes were examined by RT-PCR and Western blot assays. As shown in Figure 7*A*, mRNA levels of KRAS, NRAS, and HRAS genes remained unchanged. **MC1** (200 nM) as well as irradiation distinctly downregulated KRAS protein expression to 45% of the control. NRAS protein expression was reduced to 75% of the control, while HRAS protein expression was not significantly influenced (Fig. 7*B*). Results of Western blot assay suggested the selectivity of **MC1** against KRAS RG4 than NRAS RG4, where HRAS RNA was not reported with the presence of RG4 structure. Meanwhile, KRAS inhibition was much lower for the treatment of 200 nM **MC1** in the dark (Fig. S5), and expression of KRAS, NRAS, and HRAS was steady in **MBD**-treated tumor cells even with a high concentration of 200 nM (Fig. S6). KRAS mutant or overexpression increases the transcription of Nrf2, which is an oxidative response protein with the ability to lower intracellular ROS and permit cell survival (31, 32). Nrf2 was further detected by Western blot assay, and **MC1** revealed a dose-dependent decrease of Nrf2 expression upon irradiation. ROS production in the cells was analyzed by DCFH-DA staining, and fluorescence signals of DCF were recorded by a microscope. As presented in Figure 7*C*, fluorescence intensity was elevated dose-dependently, indicating that **MC1** effectively boosted ROS levels upon irradiation, which may lead to apoptosis. NAC, a typical ROS scavenger, was then used to prove the roles of ROS on cell survival. Growth inhibition on A549/DDP cells of **MC1**/irradiation was remarkably reversed with the presence of NAC (Fig. S7). Collectively, these findings suggested that **MC1**, when combined with irradiation, may establish a positive feedback loop that amplifies ROS production while reducing tumor cell resistance to PDT by targeting KRAS RG4.

Apoptosis was evaluated by flow cytometry and Western blot assays, and the results were presented in Figure 7, *D* and E. Upon irradiation, dose-dependent (from 0 to 200 nM) increases of early (Annexin V^+^/PI^-^) and late (Annexin V^+^/PI^+^) apoptotic populations were recorded in A549/DDP cells treated by **MC1**, as well as the increase of PARP fragment, a final executioner in apoptotic pathway. In comparison, 200 nM **MC1** could not trigger the cleavage of PARP in the dark (Fig. S5). Assessment of the cytotoxicity was finally completed in a 3D multicellular tumor spheroid (MCTS) model, which is more approximate to the condition of *in vivo* tumor growth (33). The difference of tumor volume between **MC1** group and control group was not notable after 7-days treatment. Nevertheless, the increase of red fluorescence stained by propidium iodide proved the **MC1**/irradiation-triggered cell death in MCTS (Fig. 7*F*).

### Ferroptosis and immunogenic cell death effects of MC1 in tumor cells

**Figure 8:**
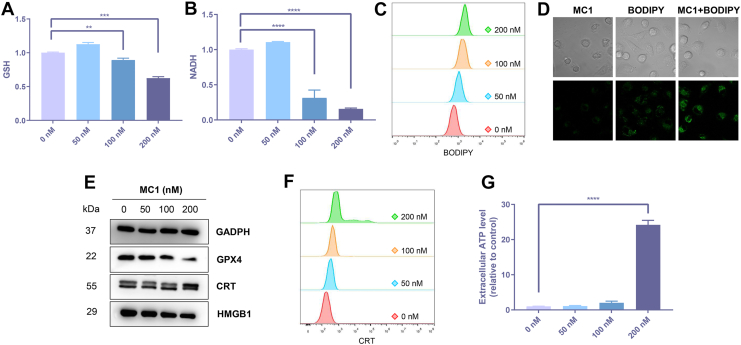
**Ferroptosis and ICD effects of MC1 in tumor cells.***A* and *B,* GSH and NADH levels of A549/DDP cells after 24-h treatment (**MC1** for 1 h, irradiation with 495 nm, 12.5 mW/cm^2^ for 15 min, and culture for another 24 h). *C* and *D,* LPO levels of A549/DDP cells after 24-h treatment, examined by flow cytometry and confocal microscopy. *E,* typical proteins of ferroptosis and ICD in A549/DDP cells after 24-h treatment, examined by Western blot. *F,* CRT expression on the surface of A549/DDP cells after 24-h treatment, examined by flow cytometry. *G,* extracellular ATP levels of A549/DDP cells after 24-h treatment. The data represent the mean ± SD (n = 3), ∗∗ for *p* < 0.01, ∗∗∗ for *p* < 0.001, ∗∗∗∗ for *p* < 0.0001 compared to the control group. ICD, immunogenic cell death; LPO, lipid peroxide; CRT, calreticulin; GSH-GPX4, glutathione peroxidase 4; HMGB1, high mobility group box 1.

GSH consumption in A549/DDP cells was determined, and **MC1**/irradiation exhibited an inhibitory effect on GSH levels in a dose-dependent manner from 0 to 200 nM (Fig. 8*A*). Photocatalytic oxidation of **MC1** was then decided by NAD^+^/NADH assay kit, showing that NADH levels in the cells were effectively decreased after drug treatment (Fig. 8*B*). In other words, **MC1** could oxidize NADH at the cellular level by irradiation, leading to the death of tumor cells.

Ferroptosis is a form of iron-dependent regulated cell death, with the accumulation of lipid peroxide (LPO) as one of the main causes (24, 34), which is closely related to the photochemical process of ROS generation (35). GSH-GPX4 (glutathione peroxidase 4) pathway is the most common upstream cascade reaction of ferroptosis, where GPX4 specifically catalyzes phospholipid and cholesterol hydroperoxide in a GSH-dependent manner (36, 37). GSH depletion is possible to inactivate GPX4, leading to ROS accumulation and lipid peroxidation (38). Besides, depletion of intracellular NADH can also be one of the characteristic events during ferroptosis (39). We first used BODIPY 581/591 C11 probe to monitor intracellular LPO levels, with the results shown in Figure 8, *C* and *D*. Green fluorescence of BODIPY 581/591 C11 in oxidation state was notably enhanced in **MC1**-treated A549/DDP cells upon irradiation, illustrated by both flow cytometry and confocal microscopy. In addition, **MC1**/irradiation-induced LPO accumulation could be reversed by ferrostatin-1, a typical ferroptosis inhibitor, further confirming the occurrence of ferroptosis (Figs. S8 and S9). GPX4 as another landmark of ferroptosis was detected by Western blot, where **MC1** triggered a dose-dependent suppression on GPX4 levels upon irradiation (Fig. 8*E*). **MC1** in the dark failed to induce ferroptosis, proved by the stable GSH level (Fig. S10), NADH level (Fig. S11), LPO level (Fig. S12), and GPX4 expression (Fig. S5) of the compound-treated tumor cells.

Ferroptosis as a nonapoptotic cell death pathway is considered as a new strategy for cancer treatment, because it displays great potential to induce immunogenic cell death (ICD) (40). Damage-associated molecular patterns are secreted and recruited to the tumor regions during ICD to remodel tumor microenvironment and provide antitumor immune responses (41, 42). Thus, three representative markers of ICD were investigated here, including calreticulin (CRT), high mobility group box 1 (HMGB1), and adenosine-5′-triphosphate (ATP). Increase of CRT expression and decrease of HMGB1 expression were recorded in **MC1**/irradiation-treated A549/DDP cells, proving the release of HMGB1 to the extracellular space (Fig. 8*E*). A more quantitative evaluation of CRT exposure was conducted by flow cytometry, and **MC1** increased the ecto-CRT (CRT exposure to the plasma membrane of viable cells) levels in a dose-dependent manner upon irradiation (Fig. 8*F*). ATP secretion to the supernatant was lastly measured by a bioluminescence detection kit, where **MC1**/irradiation caused extensive boost of extracellular ATP levels, especially with the high dose of 200 nM (Fig. 8*G*).

### Imaging and antiproliferation effects of MC1 *in vivo*

**Figure 9:**
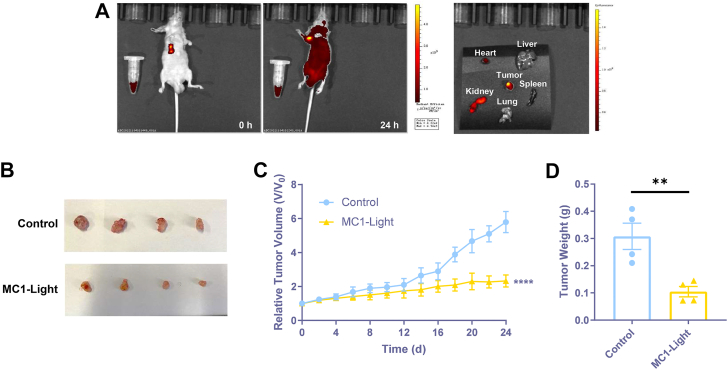
**Imaging and antiproliferation effects of MC1 *in vivo*.***A,* fluorescence images of A549/DDP-bearing mice with intratumoral injection of **MC1** (100 μM, 100 μl) for 0 h and 24 h, as well as the major organs and tumors of the mice harvested for 24 h. *B,* images of the excised tumors from A549/DDP-bearing mice treated with control, and **MC1-Light** (100 μM, 100 μl **MC1** injection as well as irradiation with 495 nm, 12.5 mW/cm^2^ for 30 min) every other day for 24 days. *C*, tumor growth curves displaying the tumor volumes measured every other day until 24 days after tumor implantation. *D,* tumor weights determined at the time of sacrifice. The data represent the mean ± SD (n = 4), ∗∗ for *p* < 0.01, ∗∗∗∗ for *p* < 0.0001 compared to the control group.

The ability of **MC1** to visualize tumor was investigated *in vivo*, where a tumor-bearing model was established by injection of A549/DDP cells to the right flanks of the nude mice. **MC1** was intratumorally injected 14 days after tumor implantation, and the fluorescence change was tracked by an animal imaging system. As shown in Figure 9*A*, the fluorescence signal was strong and limited to the tumor site right after the injection of **MC1**, suggesting that **MC1** could quickly illuminate the tumor. **MC1** was widely spread throughout the whole body after 24 h, and the fluorescence signal in the tumor was still much higher than elsewhere, proving the potential of **MC1** to be a long-term tumor visualization probe. Additionally, the corresponding imaging of major organs was performed after 24 h. Fluorescence signal was mainly distributed in the tumor, whereas there was almost no signal in the heart, liver, spleen, and lung. Fluorescence accumulation could be detected in the kidney due to its function as an important organ for drug excretion.

Antitumor effects of **MC1**/irradiation were also measured in A549/DDP-bearing nude mice, where the mice were randomly divided into two groups and treated every other day for 24 days. The mice were sacrificed, and the tumors were harvested at the end of the experiment, with the photos presented in Figures 9*B* and S13. Compared to the control group, **MC1** injection as well as irradiation with 495 nm, 12.5 mW/cm^2^ for 30 min led to significant tumor reduction. Similar results were recorded for tumor growth curves and tumor weights, with **MC1** with irradiation as a potent antitumor strategy (Fig. 9, *C* and *D*). All mice appeared healthy without visible signs of pain, distress, or discomfort during the experiment, to preliminarily infer the safety of the treatments. Body weights of the mice remained stable as shown in Figure S14. Major organs were harvested and weighed at the end of the experiment, and no significant differences of the weights could be observed between the two groups (Fig. S15). We reported that 20 mg/kg **MBD** (intraperitoneal injection) exhibited relatively weaker antitumor effects *in vivo* than **MC1** in this study (intratumoral injection plus irradiation) (16). Besides, **MBD**, rather than **MC1**, caused a continuous reduction in mouse body weights during the treatment period (Fig. S16). That is to say, the safety of **MC1** was much higher than **MBD**, since intratumoral administration of **MC1** could selectively target tumor cells than normal cells. Therefore, **MC1** could be an effective and safe agent for tumor PDT strategy.

## Conclusion

A novel small molecule (**MC1**) was discovered and explored in this study, based on our previous investigations on a typical KRAS RG4-targeted binder (**MBD**). Fluorescent response and binding selectivity of **MC1** to KRAS RG4 were proved both *in vitro* and in cells. The ability of **MC1** to visualize tumor was then determined *in vivo*, inferring its possibility to be a long-term tumor probe. More importantly, **MC1** was verified with considerable photosensitivity, demonstrating ROS production, KRAS RNA breakage, GSH consumption, and NADH oxidation both *in vitro* and in tumor cells. The irradiated **MC1** powerfully suppressed the growth of A549/DDP cells through KRAS inhibition and ROS production. Ferroptosis triggered by **MC1**/irradiation could also be recorded in tumor cells, related to GSH consumption and NADH oxidation. Finally, antitumor efficacy was determined in A549/DDP-bearing nude mice, where **MC1**/irradiation was certified as an effective and safe strategy for tumor therapy. Altogether, our study proposes a new idea for the development of KRAS RG4-targeted small molecule ligands as efficient PDT strategies for the treatment of NSCLC.

## Experimental procedures

### Synthesis and characterization

Synthesis of intermediate **4**: intermediate **3** (2.0 mmol) was mixed with 5 equiv. of *N,N′*-dimethylethane-1,2-diamine (10.0 mmol) in a round-bottomed flask. Subsequently, 2 equiv. of K_2_CO_3_ (4 mmol) and 10 ml of DMF were added to the mixture. The reaction was stirred overnight at 100 °C. After cooling, the mixture was filtered under suction to obtain an orange solid powder, which was identified as compound **4** (90% yield). Purification was deemed unnecessary. ^1^H NMR (600 MHz, CDCl_3_) δ 9.14 (d, *J* = 8.9 Hz, 2H), 7.91 (s, 2H), 7.38 (dd, *J* = 8.9, 1.6 Hz, 2H), 7.03 (s, 2H), 3.95 (d, *J* = 12.1 Hz, 4H), 3.14 (s, 6H), 3.00–2.86 (m, 6H), 1.87 (d, *J* = 12.1 Hz, 6H), 1.69–1.58 (m, 2H), 1.50 (ddd, *J* = 15.2, 12.4, 3.6 Hz, 4H), 1.06 (d, *J* = 6.4 Hz, 6H). MS (ESI) m/z: 560.3 [M + H]^+^.

Synthesis of **MC1**: Intermediate **4** (1.5 mmol) was reacted with 10 equiv. of CH_3_I (15 mmol) in 20 ml chloroform-methanol mixed solution at 60 °C overnight. Upon cooling, the reaction mixture was concentrated by removing chloroform-methanol solution using a rotary evaporator. The product was then purified by flash column chromatography using a solvent system of CH_2_Cl_2_:MeOH (20:1). After drying under reduced pressure, an orange-yellow solid powder, identified as product **MC1**, was obtained, yielding a productivity of 45%. ^1^H NMR (600 MHz, DMSO) δ 9.34 (d, *J* = 9.1 Hz, 1H), 9.02 (d, *J* = 9.0 Hz, 1H), 8.92 (d, *J* = 2.3 Hz, 1H), 8.22 (dd, *J* = 9.2, 2.4 Hz, 1H), 8.11 (d, *J* = 2.2 Hz, 1H), 7.51 (dd, *J* = 9.1, 2.3 Hz, 1H), 6.89 (d, *J* = 9.6 Hz, 2H), 4.97 (d, *J* = 12.7 Hz, 2H), 4.11 (d, *J* = 12.6 Hz, 2H), 3.99 (t, *J* = 12.8 Hz, 2H), 3.64 (s, 3H), 3.60–3.51 (m, 4H), 3.14 (s, 3H), 3.13 (s, 3H), 2.91 (td, *J* = 12.3, 2.3 Hz, 2H), 1.97 (d, *J* = 13.5 Hz, 2H), 1.91–1.79 (m, 3H), 1.69–1.59 (m, 1H), 1.40–1.27 (m, 4H), 1.01 (d, *J* = 6.6 Hz, 3H), 0.82 (d, *J* = 6.5 Hz, 3H). ^13^C NMR (151 MHz, DMSO) δ 152.19, 142.89, 142.07, 141.89, 141.23, 139.74, 138.49, 134.85, 131.94, 131.70, 130.58, 127.16, 126.23, 122.70, 120.65, 118.26, 117.03, 108.33, 102.31, 101.78, 62.59, 60.74, 49.03, 48.58, 48.38, 34.14, 30.74, 29.49, 28.64, 22.33, 21.00. HRMS (ESI) m/z: calcd for C_37_H_45_N_6_^+^ 573.3700 [M-I]^+^, found 573.3708 [M-I]^+^. Purity: 95%. The ^1^H NMR, ^13^C NMR, HRMS, and HPLC spectra of **MC1** were presented in Figures S17–S20.

### Absorption spectroscopy

Stock RNA samples were heated to 95 °C for 10 min and cooled to room temperature for G4 formation. Small aliquots of RNA samples (KRAS RG4-a and RNA HP-1, from 1 to 10 μM) were added to **MC1** (5 μM) in 10 mM Tris-HCl buffer (pH 7.4, containing 100 mM KCl), and UV-Vis absorption spectra were performed on an Agilent Cary 60 UV-Vis Spectrophotometer (Agilent Technologies) at 400∼700 nm.

### Fluorescence spectroscopy

Small aliquots of RNA samples (KRAS RG4-a and RNA HP-1, from 1 to 10 μM) were added to **MC1** (5 μM) in 10 mM Tris-HCl buffer (pH 7.4, containing 100 mM KCl), and fluorescence spectra excited at 480 or 600 nm were performed on a FluoroMax-4 spectrophotometer (HORIBA) with 2 mm × 10 mm path length at 3 nm excitation and emission slit widths. Fluorescence spectra of 5 μM **MC1** and 10 μM RNA samples (KRAS RG4-a, KRAS RG4-b, KRAS RG4-c, KRAS RG4-long, RNA HP-1, and RNA HP-2) were also recorded excited at 480 or 600 nm. For fluorescence competitive assay, small aliquots of RNA HP-1 (from 5 to 20 μM) were added to the solution containing 5 μM **MC1** and 10 μM KRAS RG4-a.

### CD studies

KRAS RG4-a (5 μM) was incubated with **MC1** (25 μM) 10 mM Tris-HCl buffer with 100 mM KCl. Subsequently, samples were irradiated with LED light (495 nm, 12.5 mW/cm^2^) when required. CD spectra for all samples were recorded in the range of 220 to 300 nm. For CD melting assay, the procedure was similar except for the concentration of KCl at 10 mM.

### Confocal laser scanning microscopy

The authenticity of A549/DDP cell line (Procell) was validated using STR profiling, and the cell line was tested free from *mycoplasma* contamination.

Live A549/DDP cells were stained with **MC1** (2 μM), MitoTracker Red, and DAPI for 20 min. Images were captured by a LSM 880 laser scanning confocal microscope (Zeiss, Germany). For displacement assay, live A549/DDP cells were incubated with 10 μM DMS for 30 min and then added with 2 μM **MC1** for another 30 min. For digesting assay, before stained with **MC1**, the cells were treated with Triton-X 100 (0.1%, 37 °C, 30 min) and then DNase I or RNase A (100 U/ml, 37 °C, 2 h).

### ROS analysis

For *in vitro* ROS analysis, 10 μM **MC1** was incubated with 10 μM RNA samples (KRAS RG4-a, KRAS RG4-b, RNA HP-1, and ssRNA) in 10 mM Tris-HCl buffer (pH 7.4, containing 100 mM KCl), as well as DCFH. The mixtures were then irradiated with a green LED light (495 nm, 12.5 mW/cm^2^) for 0, 5, 10, 15, 20, 25, and 30 min, and fluorescence intensity was recorded by the fluorescence microplate reader (BioTek).

For ROS analysis in tumor cells, A549/DDP cells were treated with **MC1** (0, 50, 100, and 200 nM) for 1 h and washed with PBS, followed by irradiation with a green LED light (495 nm, 12.5 mW/cm^2^) for 15 min and culture for another 24 h. Next, cells were incubated with DCFH-DA for 30 min at 37 °C in the dark. Green fluorescence was assessed using the fluorescence microscope (Leica).

### Electrophoretic mobility shift assay

**MC1** (10 μM) was incubated with 10 μM RNA samples (KRAS RG4-a and ssRNA) in 10 mM Tris-HCl buffer (pH 7.4, containing 100 mM KCl). The mixtures were then irradiated with a green LED light (495 nm, 12.5 mW/cm^2^) for 0, 0.5, 1, 2, and 3 h and separated on 15% native polyacrylamide gel in TBE running buffer at 60 V for 2 h. The gel was stained by EB and image by GelView 1500 Pro (BLT).

### GSH analysis

For *in vitro* GSH analysis, 20 μM **MC1** was incubated with 200 μM GSH, followed by irradiation with a green LED light (495 nm, 12.5 mW/cm^2^) for different time points. The mixture was then added with 20 μg/ml 5,5′-dithiobis-(2-nitrobenzoic acid), and the absorption spectroscopy was recorded at 350 to 550 nm. For GSH analysis in tumor cells, A549/DDP cells were treated with **MC1** and irradiation as mentioned above. GSH levels were determined by GSH and GSSG Assay Kit (Beyotime), according to the supplier's manual. Relative GSH levels were corrected by the cell viability and demonstrated by the ratios to control group.

### NADH analysis

For *in vitro* NADH analysis, 20 μM **MC1** was incubated with 200 μM NADH, followed by irradiation with a green LED light (495 nm, 12.5 mW/cm^2^) for different time points, and the absorption spectroscopy was recorded at 230 to 400 nm. For NADH analysis in tumor cells, A549/DDP cells were treated with **MC1** and irradiation as mentioned above. NADH levels were determined by NAD^+^/NADH Assay Kit (Beyotime), according to the supplier's manual. Relative NADH levels were corrected by the cell viability and demonstrated by the ratios to control group.

### Dual luciferase reporter assay

PsiCHECK-2 vector (Promega) was inserted with a wildtype or mutant 5′-UTR sequence of KRAS containing G4 structure (sequences shown in Table S2). After the transfection of the plasmids into HEK293 cells, **MC1** was added to the cells and incubated for 24 h. Luciferase activity was measured with Dual-Luciferase Reporter Assay System (Promega), according to the supplier's manual, on a Synergy H1 multimode microplate reader (BioTek). RL activity was normalized by FL activity.

### Cytotoxicity assay

A549/DDP cells were cultured in the 96-well plate with the density of 5000 cells/well and treated with **MC1** for 1 h and washed with PBS, followed by irradiation with an LED light (495 nm or 595 nm, 12.5 mW/cm^2^) for 15 min and culture for another 48 h. Cells were incubated with CCK-8 solution for 2 h, and absorbance values at 450 nm were measured with the spectrophotometer (BioTek). IC_50_ values were determined by plotting cell viability *versus* drug dose.

### RT-PCR assay

A549/DDP cells were treated with **MC1** (0, 50, 100, and 200 nM) for 1 h and washed with PBS, followed by irradiation with a green LED light (495 nm, 12.5 mW/cm^2^) for 15 min and culture for another 24 h. Total RNA of the cells was extracted with Cell Total RNA Isolation Kit (Foregene), according to the supplier's manual. The concentration of RNA was quantitated by a NanoDrop 1000 spectrophotometer (Thermo Scientific). Reverse transcription was completed with Transcriptor First Strand cDNA Synthesis Kit (Roche), according to the supplier's manual, to harvest cDNA. Afterward, PCR was performed on a PCR device (Thermo Scientific), and the program for all genes included a denaturing cycle (95 °C for 5 min) and 28 PCR cycles (95 °C for 30 s, 58 °C for 30 s and 72 °C for 40 s). Sequences of the PCR primers were listed in Table S3. PCR products were confirmed with agarose gel electrophoresis and SuperRed staining.

### Western blot assay

Total protein of **MC1**/irradiation-treated A549/DDP cells was extracted with RIPA buffer containing protease inhibitors for 30 min at 4 °C. The concentration of protein was quantitated with Pierce BCA Protein Assay Kit (Thermo Scientific), according to the supplier's manual. Twenty microgram protein was separated by SDS-PAGE and transferred to PVDF membrane (Bio-Rad). The membrane was blocked in 5% skim milk for 1 h at room temperature and incubated with the primary antibodies overnight at 4 °C and the peroxidase-conjugated secondary antibodies for 1 h at room temperature. Information of the antibodies was listed in Table S4. Finally, protein bands were detected by Enhanced Chemiluminescence Kit (Bio-Rad) and captured by an imaging system (CLiNX, China). Quantifications of the blots were calculated in gray scale by Image J software.

### Apoptosis analysis

**MC1**/irradiation-treated A549/DDP cells were collected and stained by Annexin V-633 Apoptosis Detection Kit (Dojindo), according to the supplier's manual. Apoptosis signals were recorded by an Attune N x T Flow Cytometer (Thermo Scientific).

### MCTS formation assay

A549/DDP cells were cultured in the 96-well round bottom ultra-low attachment plate (Corning) with the density of 2000 cells/well. MCTS aggregates were formed after 5 days and then treated by **MC1** as well as irradiation for 7 days. Viability of MCTS were determined by Calcein AM/PI Double Staining Kit (Dojindo), according to the supplier's manual.

### LPO assay

**MC1**/irradiation-treated A549/DDP cells were collected and stained by Lipid Peroxidation Assay Kit with BODIPY 581/591 C11 (Beyotime), according to the supplier's manual. Fluorescence signals of oxidized BODIPY 581/591 C11 were recorded by an Attune N x T Flow Cytometer (Thermo Scientific) or a LSM 880 laser scanning confocal microscope (Zeiss).

### ICD analysis

For CRT analysis, **MC1**/irradiation-treated A549/DDP cells were collected and stained by rabbit anti-human Calreticulin monoclonal antibody (1:200 dilution) (Abcam) for 30 min. Then, cells were stained by secondary Alexa Fluor 647-labeled anti-rabbit IgG antibody (1:2000 dilution) (Abcam) for 30 min, and analyzed by an Attune N x T Flow Cytometer (Thermo Scientific). For ATP analysis, the culture media of **MC1**/irradiation-treated A549/DDP cells were collected and analyzed by ENLITEN ATP Assay System Bioluminescence Detection Kit (Promega), according to the supplier's manual. Relative ATP levels were corrected by the cell viability and demonstrated by the ratios to control group.

### *In vivo* tumor imaging and antitumor study

Four-week-old male BALB/c nude mice were housed in a specific pathogen-free condition with a 12 h light/dark cycle and fed with water and food ad libitum. Each mouse was injected subcutaneously to the right flank with 1 × 10^7^ A549/DDP cells mixed with Matrigel. For tumor imaging, 14 days after tumor implantation, **MC1** (100 μM, 100 μl) was injected into the tumor sites, and the mice were imaged by an Animal Imaging System (Caliper Life Science). Major organs and tumors of the mice were also harvested and imaged.

For antitumor study, after tumor volumes reached approximate 50 mm^3^, the mice were randomly divided into two groups and treated with saline and **MC1-Light** (100 μM, 100 μl **MC1** injection as well as irradiation with 495 nm, 12.5 mW/cm^2^ for 30 min) every other day. Tumor sizes and body weights were measured every other day, and tumor volumes were calculated as half × length × width (2). After drug treatment for 24 days, the mice were sacrificed, and the tumors and the major organs (hearts, livers, spleens, lungs, and kidneys) were removed, photographed, and weighed. The animal experiments were approved by the Animal Ethics Committee of Shenzhen University.

### Statistics

The data were plotted and expressed as the mean ± SD, for at least three independently performed experiments. The data were statistically analyzed using one-way ANOVA with Tukey's *post hoc* test, and the value of *p* < 0.05 was statistically significant.

## Data availability

Data are available from the authors upon request.

## Conflict of interest

The authors declare that they have no conflicts of interest with the contents of this article.
