# Supplementary material for: Photodynamic activation of a KRAS RNA G-quadruplex–targeted photosensitizer induces ferroptosis in cisplatin-resistant non–small cell lung cancer
Source: J Biol Chem. 2026 Jan 20;302(3):111181. doi: 10.1016/j.jbc.2026.111181 (PMC12914405; doi:10.1016/j.jbc.2026.111181)
Supplement: Supplementary Data [file mmc1.docx]

**Supporting Information**

**Photodynamic activation of a KRAS RNA G-quadruplex–targeted photosensitizer induces ferroptosis in cisplatin-resistant non-small cell lung cancer**

Xiao-Dong Wang, Jia-Hong Lin, Ming-Hao Hu*

Nation-Regional Engineering Lab for Synthetic Biology of Medicine, International Cancer Center, School of Pharmacy, Shenzhen University Medical School, Shenzhen 518060, China

* Corresponding author, humhao1229@szu.edu.cn

**Table of Contents:**

| **Figure S1.** Fluorescence spectra of **MBD** titrated with KRAS RG4-a. | S2 |
| --- | --- |
| **Figure S2.** CD melting assay of KRAS RG4-a without or with the presence of the compounds. | S2 |
| **Figure S3.** Fluorescence spectra of **MC1** with the presence of different RG4s. | S2 |
| **Figure S4.** CD spectra of **MC1** and KRAS RG4-a with irradiation. | S3 |
| **Figure S5.** Changes of typical proteins by **MC1** with or without irradiation in A549/DDP cells. | S3 |
| **Figure S6.** Expression of RAS-related proteins induced by **MBD** in A549/DDP cells. | S3 |
| **Figure S7.** Cytotoxicity of **MC1** as well as irradiation in A549/DDP cells, with the presence of NAC. | S4 |
| **Figure S8.** LPO levels induced by **MC1** as well as irradiation in A549/DDP cells, with the presence of Fer-1, examined by flow cytometry. | S4 |
| **Figure S9.** LPO levels induced by **MC1** as well as irradiation in A549/DDP cells, with the presence of Fer-1, examined by confocal microscopy. | S4 |
| **Figure S10.** GSH levels induced by **MC1** with or without irradiation in A549/DDP cells. | S5 |
| **Figure S11.** NADH levels induced by **MC1** with or without irradiation in A549/DDP cells. | S5 |
| **Figure S12.** LPO levels induced by **MC1** with or without irradiation in A549/DDP cells. | S5 |
| **Figure S13.** Images of tumors in control, and **MC1-Light** groups. | S6 |
| **Figure S14.** Body weights of mice in control, and **MC1-Light** groups. | S6 |
| **Figure S15.** Weights of the major organs of mice in control, and **MC1-Light** groups. | S6 |
| **Figure S16.** Body weight changes of A549/DDP-bearing mice in **MBD**, and **MC1-Light** groups. | S7 |
| **Figure S17–20.** ^1^H NMR, ^13^C NMR, HRMS and HPLC spectra of **MC1**. | S8 |
| **Table S1.** Sequences of the oligonucleotides used in this study. | S10 |
| **Table S2.** Inserted sequences to the dual-luciferase reporter plasmid. | S10 |
| **Table S3.** Primers used in RT-PCR assay. | S10 |
| **Table S4.** Antibodies used in western blot assay. | S11 |

**
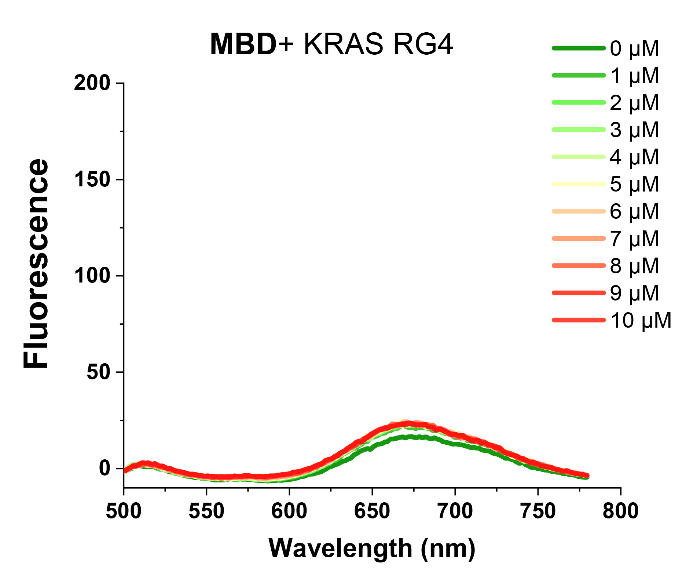
**

**Figure S1.** Fluorescence spectra of 5 μM **MBD** titrated with KRAS RG4-a from 0 to 10 μM.

**
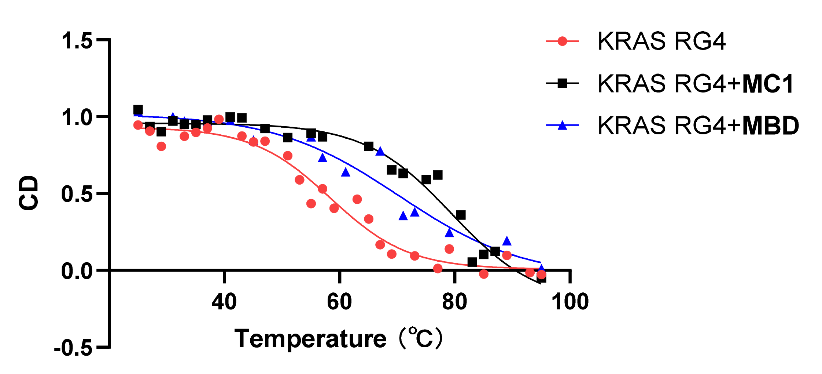
**

**Figure S2.** CD melting assay of KRAS RG4-a (5 μM) without or with the presence of the compounds (25 μM).





**Figure S3.** Fluorescence spectra of **MC1** (5 μM) with the presence of KRAS RG4-long, KRAS RG4-a and NRAS RG4 (10 μM).


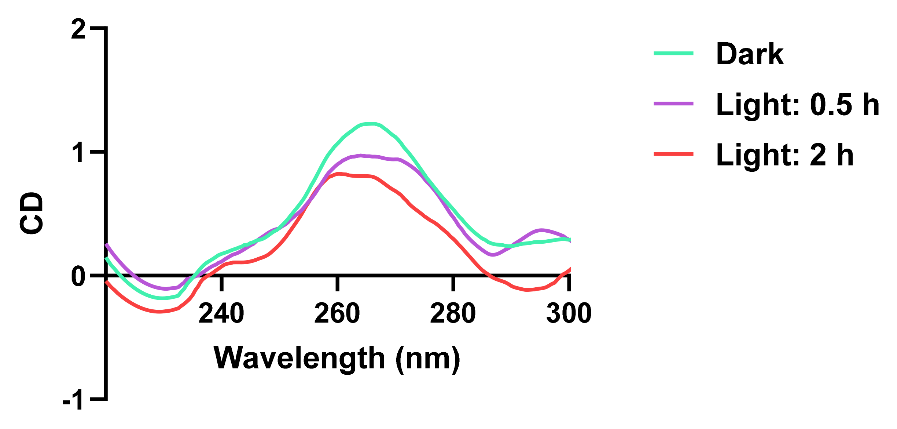


**Figure S4.** CD spectra of **MC1** and KRAS RG4-a irradiated with 495 nm, 12.5 mW/cm^2^ for different time points.

**
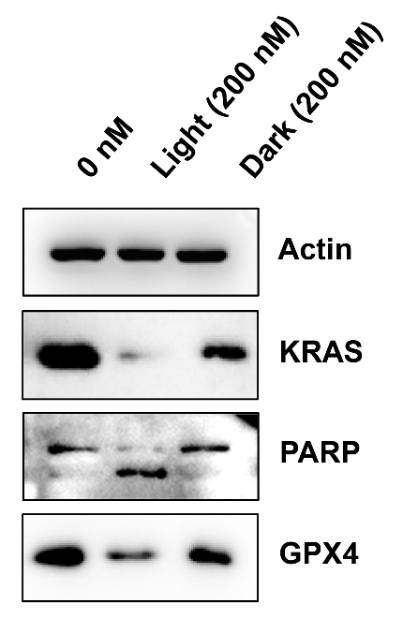
**

**Figure S5.** Changes of typical proteins induced by **MC1** with or without irradiation in A549/DDP cells.

**
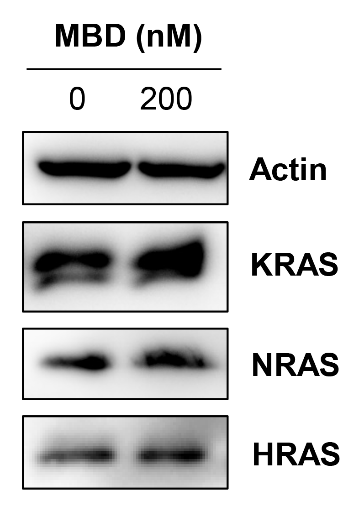
**

**Figure S6.** Expression of RAS-related proteins induced by **MBD** in A549/DDP cells.

**
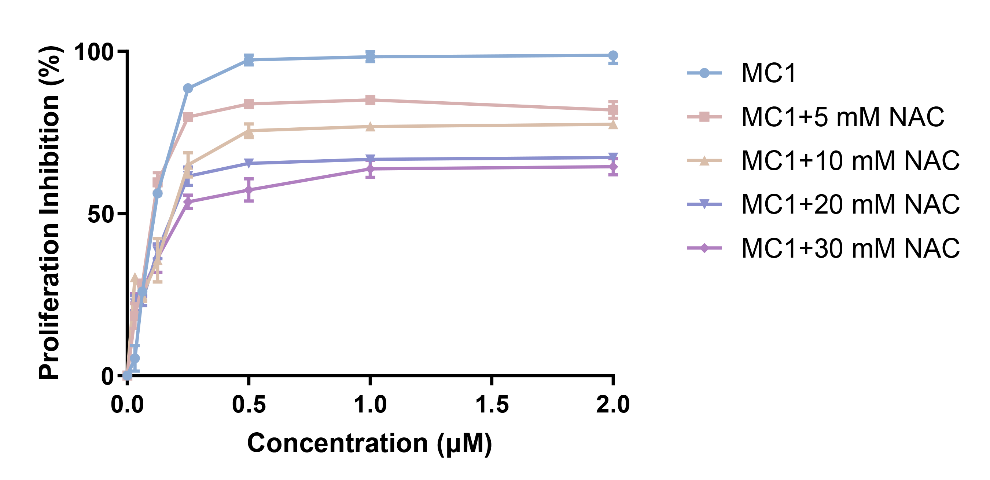
**

**Figure S7.** Cytotoxicity of **MC1** as well as irradiation in A549/DDP cells, with the presence of NAC.

**
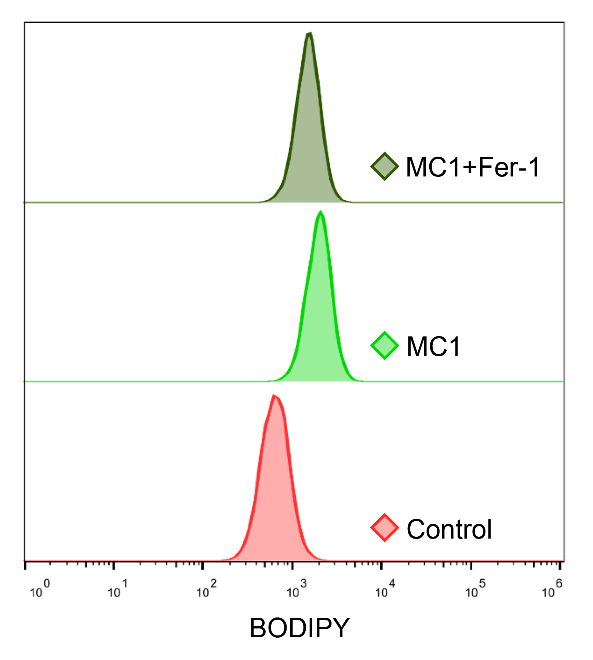
**

**Figure S8.** LPO levels induced by **MC1** as well as irradiation in A549/DDP cells, with the presence of Fer-1, examined by flow cytometry.

**
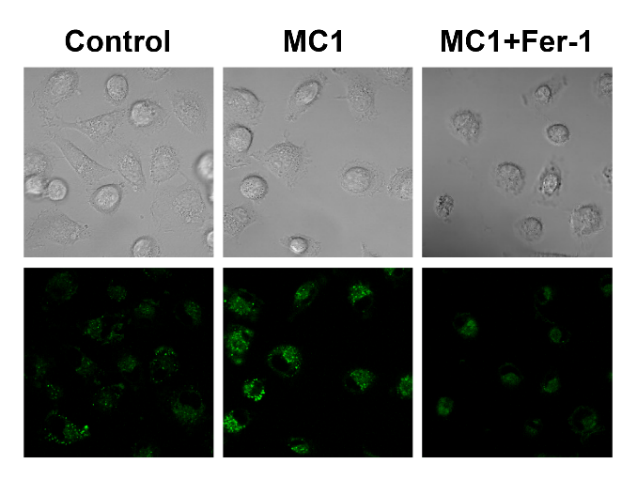
**

**Figure S9.** LPO levels induced by **MC1** as well as irradiation in A549/DDP cells, with the presence of Fer-1, examined by confocal microscopy.

**
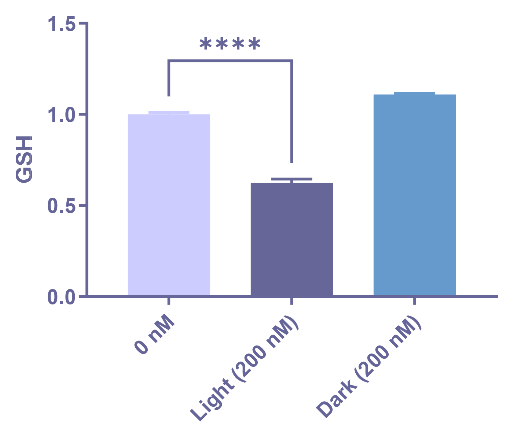
**

**Figure S10.** GSH levels induced by **MC1** with or without irradiation in A549/DDP cells.

**
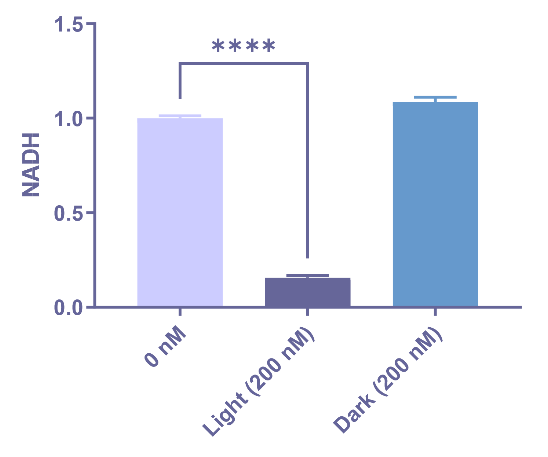
**

**Figure S11.** NADH levels induced by **MC1** with or without irradiation in A549/DDP cells.

**
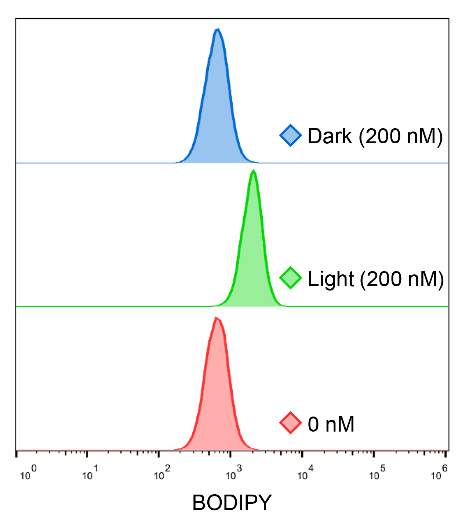
**

**Figure S12.** LPO levels induced by **MC1** with or without irradiation in A549/DDP cells.

**
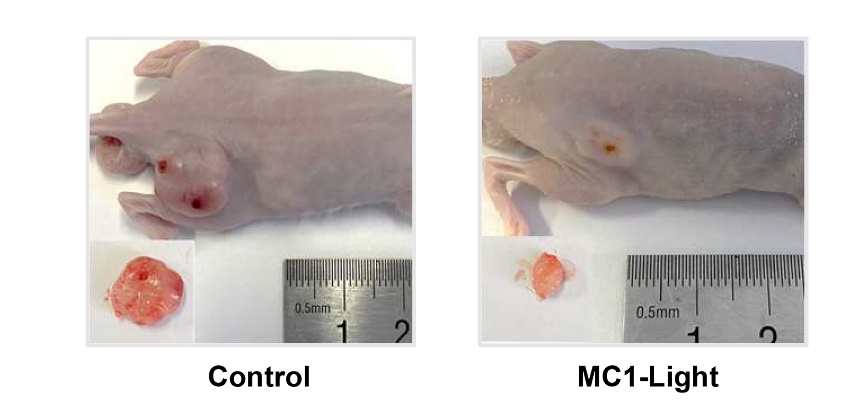
**

**Figure S13.** Representative images of A549/DDP-bearing mice and tumors in control, and **MC1-Light** groups.

**
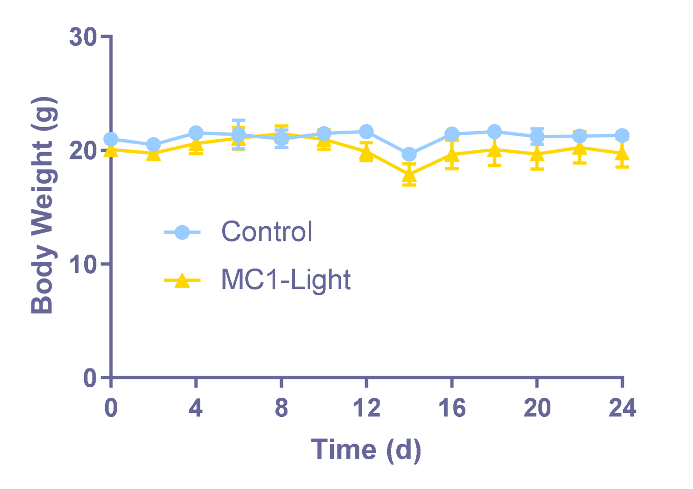
**

**Figure S14.** Body weights of A549/DDP-bearing mice in control, and **MC1-Light** groups.

**
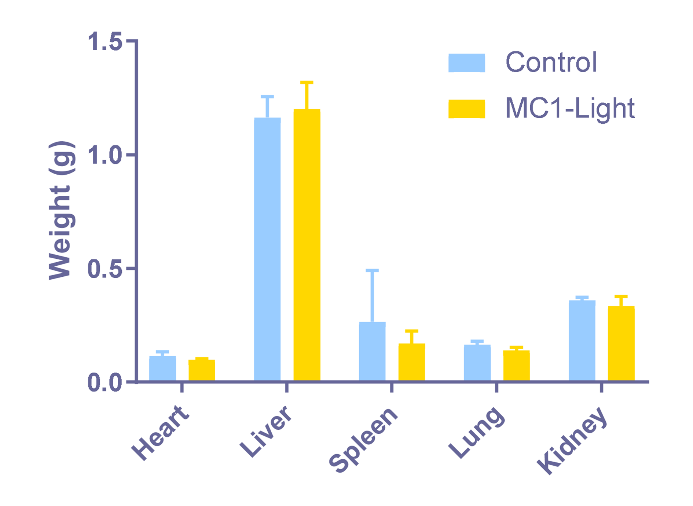
**

**Figure S15.** Weights of the major organs of A549/DDP-bearing mice in control, and **MC1-Light** groups.


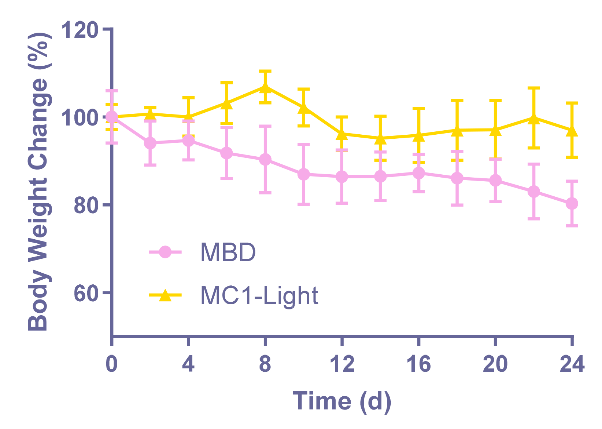


**Figure S16.** Body weight changes of A549/DDP-bearing mice in **MBD**, and **MC1-Light** groups.

**
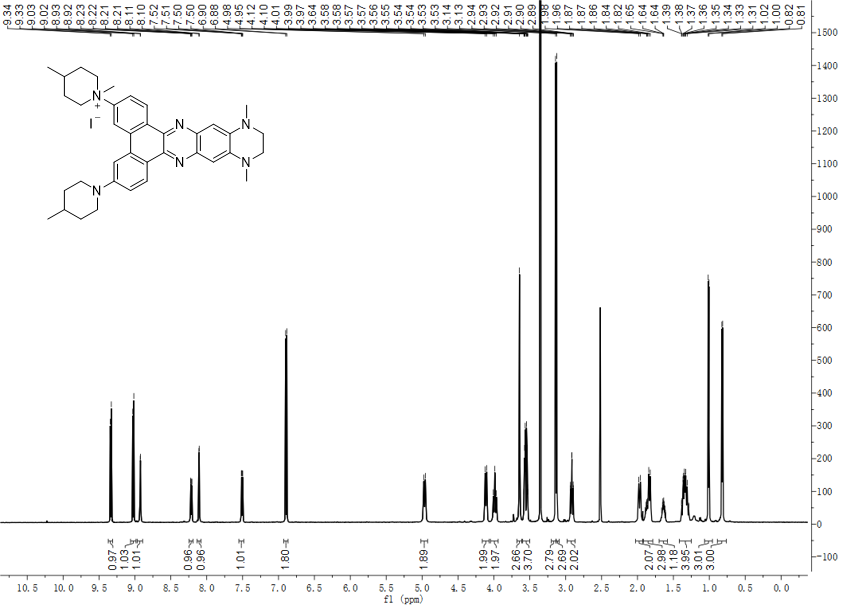
**

**Figure S17.** ^1^H NMR spectrum of **MC1**


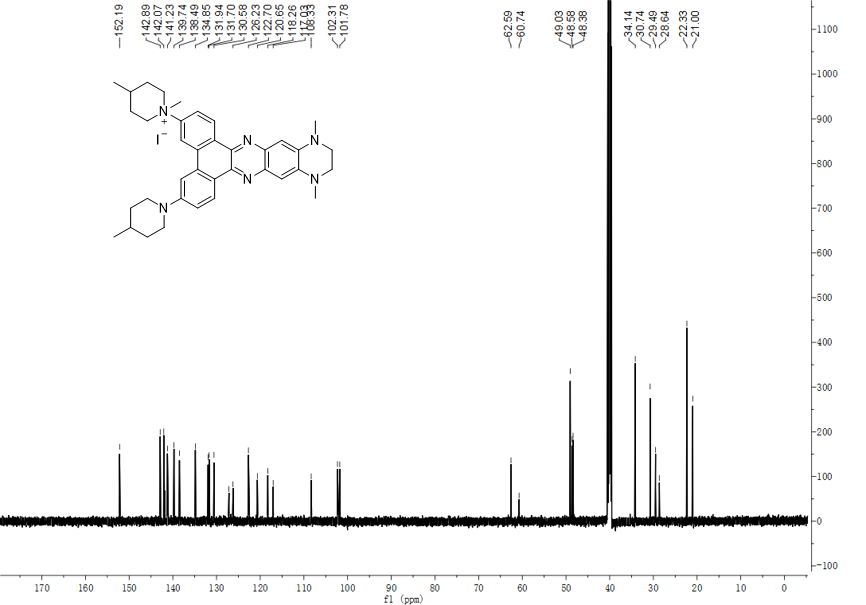


**Figure S18.** ^13^C NMR spectrum of **MC1**

**
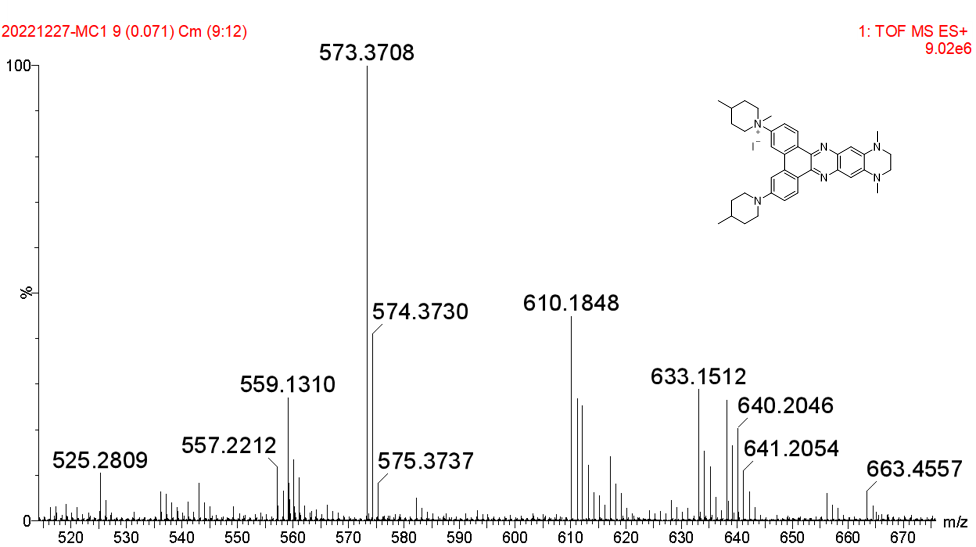
**

**Figure S19.** HRMS spectrum of **MC1**

**
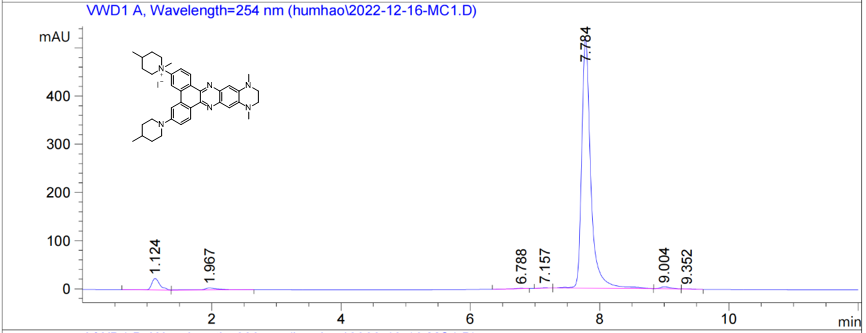
**

**Figure S20.** HPLC spectrum of **MC1**

**Table S1.** Sequences of the oligonucleotides used in this study.

| Name | Sequence (5’→3’) |
| --- | --- |
| KRAS RG4-a | GCGGCGGCGGAGGCAGCA |
| KRAS RG4-b | GGCGGCGGCAGUGGCGGCGG |
| KRAS RG4-c | AAGGUGGCGGCGGCUCG |
| KRAS RG4-long | GCGGCGGCGGAGGCAGCAGCGGCGGCGGCAGUGGCGGCGGCGAAGGUGGCGGCGGC |
| RNA HP-1 | GGCCGCCGCAGUGGCGGCGG |
| RNA HP-2 | CAGUACAGAUCUGUACUG |
| ssRNA | UGUAAACAUCCUACACUCAGCU |

**Table S2.** Inserted sequences to the dual-luciferase reporter plasmid.

| Name | Sequence (5’→3’) |
| --- | --- |
| KRAS 5’-UTR  wild type | TCCTAGGCGGCGGCCGCGGCGGCGGAGGCAGCAGCGGCGGCGGCAGTGGCGGCGGCGAAGGTGGCGGCGGCTCGGCCAGTACTCCCGGCCCCCGCCATTTCGGACTGGGAGCGAGCGCGGCGCAGGCACTGAAGGCGGCGGCGGGGCCAGAGGCTCAGCGGCTCCCAGGTGCGGGAGAGAGGCCTGCTGAAA |
| KRAS 5’-UTR  mutant | TCCTAGGCGGCGGCCGCAACAACAAAAACAGCAGCAACAACAACAATAACAACAACGAAAATAACAACAACTCGGCCAGTACTCCCGGCCCCCGCCATTTCGGACTGGGAGCGAGCGCGGCGCAGGCACTGAAGGCGGCGGCGGGGCCAGAGGCTCAGCGGCTCCCAGGTGCGGGAGAGAGGCCTGCTGAAA |

**Table S3.** Primers used in RT-PCR assay.

| Gene | Forward primer (5’→3’) | Reverse primer (5’→3’) |
| --- | --- | --- |
| β-actin | GCATCCTGTCGGCAATGC | GTTGCTATCCAGGCTGTGC |
| KRAS | CGAATATGATCCAACAATAGAG | ATGTACTGGTCCCTCATT |
| NRAS | CCTATACAATGTATGTAATTTGTTTCC | CAATGCACCAAAGTTTTACAATATTTGAAC |
| HRAS | TTTGTGGACGAGTATGATCCCA | TGCTCCCTGTACTGATGGATG |

**Table S4.** Antibodies used in western blot assay.

| Antibody | Source | Cat# | Dilution |
| --- | --- | --- | --- |
| NRAS | Abcam | ab198820 | 1:1000 |
| KRAS | Abcam | ab275876 | 1:1000 |
| HRAS | Abcam | ab32417 | 1:1000 |
| Nrf2 | CST | 12721 | 1:1000 |
| PARP | CST | 9532 | 1:1000 |
| Cleaved PARP | CST | 9541 | 1:1000 |
| GPX4 | CST | 59735 | 1:1000 |
| CRT | CST | 12238 | 1:1000 |
| HMGB-1 | CST | 3935 | 1:1000 |
| GAPDH | CST | 5174 | 1:1000 |
| β-ACTIN | CST | 4970 | 1:1000 |
